# Clinical features and genetic analysis of a family with t(5;9) (p15;p24) balanced translocation leading to Cri-du-chat syndrome in offspring

**DOI:** 10.3389/fgene.2025.1550937

**Published:** 2025-05-08

**Authors:** Jing Zhao, Ping Chen, Yijia Ren, Shurong Li, Weiyi Zhang, Yan Li, Fengyu Wang

**Affiliations:** ^1^ Department of Obstetrics and Gynecology, The First Affiliated Hospital of Henan University of Chinese Medicine, Zhengzhou, Henan, China; ^2^ Zhengzhou KingMed Clinical Laboratory Co., Ltd, Zhengzhou, Henan, China; ^3^ Department of Neurology, Henan Provincial People’s Hospital, People’s Hospital of Zhengzhou University, Zhengzhou, Henan, China

**Keywords:** balanced translocation family, characteristics, Cri-du-chat syndrome, prenatal diagnosis, genetic counseling

## Abstract

**Background:**

Balanced translocations are common chromosomal structural abnormalities that usually do not involve a gain or loss of genetic material; and carriers usually display normal phenotypes and intelligence. However, unbalanced rearranged gametes can be produced during the meiotic division of reproductive cells, leading to infertility, miscarriage, stillbirth, or the birth of newborns with malformations and chromosomal abnormalities. These adverse pregnancy outcomes create significant burdens for families and societies and affect the quality of life of newborns, threatening their health.

**Objective:**

Individuals carrying balanced translocations can have phenotypically normal offspring, but they also face high risks of natural miscarriage and giving birth to newborns with chromosomal abnormalities. We characterized individual clinical features and conducted a genetic analysis of the members of a family with t (5; 9) (p15; p24) balanced translocation leading to Cri-du-chat syndrome in the offspring.

**Study design:**

We performed a chromosomal karyotyping with high-resolution G-banding on the proband and her family members to detect their chromosomal configurations. We also used chromosomal microarray analyses (CMA) to detect copy number variants. Enrichment analysis of genes in the duplicated or deleted regions of 5p and 9p was performed using Metascape. Results: The proband (III7), her father (II3), her brother (III5), and her cousin (III14) were all carriers of the t (5; 9) (p15; p24) balanced translocation. Offspring (IV5, IV7, IV9, and IV12) were affected. Genetic microarray results of IV7 showed a 26.3 Mb deletion on chromosome five and a 15.3 Mb duplication on chromosome 9. Genetic microarray results of IV5, IV9, and IV12 showed a 26.5 Mb duplication on chromosome five and a 15.4 Mb deletion on chromosome 9.

**Conclusion:**

This study reports a rare familial balanced translocation pedigree, particularly noting that the offspring can suffer from Cri-du-chat syndrome, which suggests a potential new genetic model for this syndrome. It provides novel insights for genetic counseling and prenatal diagnosis in patients with adverse pregnancy outcomes before attempting another pregnancy.

## 1 Introduction

In 1963, the French scientist Lejeune first described the Cri-du-chat syndrome, naming it after the characteristic cat-like cry of the affected newborns. The syndrome is a rare genetic disorder caused by a terminal deletion on the short arm (5p) of chromosome 5 (Van Buggenhout et al., 2000). CdCS, also known as 5p deletion syndrome, is caused by partial or total deletion of the short arm of chromosome 5, with main clinical features including intrauterine growth retardation, hypotonia, intellectual disability, characteristic cat-like cry (may disappear with age), microcephaly, and distinctive facial features. Its incidence ranks first among the autosomal structural abnormalities (approximately one in 15,000 to one in 50,000) accounting for 1.3% of all the pediatric chromosomal diseases. Literature reports indicate that 10%–15% of the cases of Cri-du-chat syndrome originate from a balanced translocation in a parent, with most deletions of 5p being paternal in origin, resulting in multiple malformations and severe intellectual disabilities in their offspring (Tyagi et al., 2010). We combined G-banded chromosomal karyotyping and chromosomal microarray analysis (CMA) to conduct a genetic analysis on members of a family with a balanced translocation leading to Cri-du-chat syndrome in the offspring. Our findings expand the spectrum of pathogenic variations associated with Cri-du-chat syndrome, deepen the understanding of the disease, and provide a basis for genetic counseling and prenatal diagnosis for members of translocation-carrying families.

## 2 Materials and methods

### 2.1 Patients

We selected a carrier of the t (5; 9) (p15; p24) balanced translocation and her family (a total of 12 members) who visited our hospital in March 2023. The Ethics Committee of the hospital reviewed and approved the study, and all participants or their guardians signed informed consent forms.

### 2.2 Methods

#### 2.2.1 Collection of clinical data

We collected clinical data of the family of the pregnant woman carrying the t (5; 9) (p14; p22) balanced translocation, including medical and family histories, and admission physical examination and laboratory tests results.

#### 2.2.2 Chromosomal karyotype analysis

Peripheral blood samples (3–5 mL) were collected from each patient using sodium heparin anticoagulant tubes. Volumes from 0.3 mL to 0.5 mL were inoculated into 5–7 mL of peripheral blood lymphocyte culture medium. Subsequently, slides were prepared for examination after hypotonic treatment, fixation, aging, banding, and staining. We analyzed the slides using an automated scanning microscope for karyotype analysis.

#### 2.2.3 CMA testing for individuals with chromosomal abnormalities

Peripheral blood samples (2 mL) and amniotic fluid samples were collected from the study participants and sent for testing. We performed quality controls of the samples using quantitative fluorescence polymerase chain reaction (PCR) to rule out maternal cell contamination. Chromosomal microarray analyses (CMAs) were conducted using the CytoScan750K chip (Affymetrix, United States) following the reagent and instrument manual instructions. The processes included quality control, digestion, PCR, purification, labeling, hybridization, and scanning.

#### 2.2.4 Pathogenicity analysis

We annotated and analyzed the copy number variants (CNVs) on the basis of data on the Database of Genomic Variants (DGV [http://dgv.tcag.ca/]), the Online Mendelian Inheritance in Man (OMIM [https://www.omim.org/]), and PubMed (https://pubmed.ncbi.nlm.nih.gov/). Additionally, we analyzed the clinical significance of CNVs by referring to the ClinGen clinical genome resource center (http://www.ncbi.nlm.nih.gov/projects/dbvar/clingen/). We followed the guidelines issued jointly by the American College of Medical Genetics and Genomics (ACMG) and ClinGen to analyze the pathogenicity of the detected variants.

#### 2.2.5 Enrichment analysis of gene functions

Enrichment analysis of Metascape database Metascape (http://metascape.org/) is a powerful tool for gene function annotation analysis, using Metascape to perform enrichment analysis on 60 OMIM genes including *TRIO* and *CTNND2* in the 5p15.33p14.1 region, and 45 OMIM genes including *NFIB* and *SMARCA2* in the 9p24.3-p22.3 region.

## 3 Results

### 3.1 Clinical data

#### 3.1.1 Medical and family history

The proband (III7), a 35-year-old woman, presented to our hospital’s outpatient department for pre-pregnancy examination and genetic counseling due to a history of repeated adverse pregnancy outcomes. Her obstetric history included four pregnancies and three live births. In 2009, she delivered a female newborn (IV6) at a local hospital. The newborn had a weak cry resembling a cat’s meow and died after 7 days at the local hospital from congenital pneumonia and high fever. In 2010, the proband delivered a male infant (IV7) at a local health center, he also had a cat-like cry at birth. The boy, a 13-year-old at the time of writing, could only consume liquid foods, had low muscle tone in the arms and legs, could not walk independently, and presented severe intellectual disability (he was unable to communicate). Genetic testing confirmed a diagnosis of Cri-du-chat syndrome. In 2013, the proband delivered another male infant (IV8) via cesarean section at a local hospital. The baby weighed 10.5 pounds and was transferred to Zhengzhou Children’s Hospital due to high fever and atelectasis, but died 1 day later suspected of presenting also Cri-du-chat syndrome. In 2019, the proband underwent amniocentesis at 16 + weeks of gestation, and genetic testing on IV9 revealed abnormal CNVs, leading to a medical termination. The proband had no significant history of diseases during pregnancy and no history of exposure to radiation or toxic substances.

**FIGURE 1:**
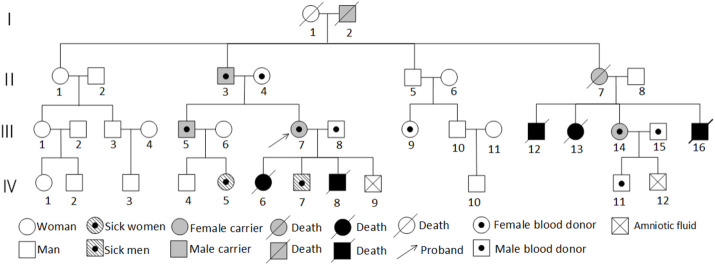
Pedigree of the family of a carrier of a t (5; 9) (p15; p24) balanced translocation.

Further inquiry into the family history revealed that the proband’s uncle’s children had all died young, but chromosomal testing was not performed due to limitations. The proband’s maternal family had no history of adverse pregnancy outcomes, suggesting a possible paternal inheritance, with the proband’s father being a carrier. The proband’s brother (III5) was a normal-looking man with normal intelligence. In 2018, he fathered a healthy male newborn (IV4); and, in 2021, he fathered a female newborn (IV5) with distinctive facial features, intellectual disability, a speech impairment, and developmental delays. The proband’s second aunt (II7) had normal intelligence but died during childbirth. She had given birth to several full-term infants (III12, III13, III16) who died, leading to speculation that II7 might have been a carrier or affected individual. However, one daughter survived, the proband’s female cousin (III14), who had a normal appearance, normal intelligence, and a history of two pregnancies and one birth. In 2017, she underwent amniocentesis at 20 + weeks of gestation, and genetic testing on IV12 revealed abnormal CNVs, leading to a medical termination. This cousin had one surviving male infant (IV11) without phenotypic or genetic abnormalities. III14 was the only cousin of the proband from that aunt, who survived and had a normal appearance and normal intelligence. III14’s obstetric history included a normal male infant born in 2012 without apparent phenotypic or genetic abnormalities; amniocentesis at 20 + weeks of gestation in 2017 revealed CNV abnormalities in IV12, leading to a medical termination. Figure 1 shows the family diagram.

#### 3.1.2 Physical examination upon admission

The proband (III7) was a normal-looking, intelligent, communicative woman, 160-cm tall and weighing 61 kg. Routine blood tests, liver and kidney function tests, a coagulation profile, and other biochemical indicators were all normal. Screening for TORCH infections and tests related to recurrent miscarriage, including thrombophilia, antiphospholipid syndrome, and rheumatoid arthritis also revealed absence of significant abnormalities. Ultrasound scans of the heart, liver, gallbladder, spleen, and pancreas were normal. There were no viral infections during pregnancy, nor exposure to toxic substances or radiation. The proband’s husband was phenotypically normal, denied consanguinity, and his paternal grandparents also presented normal phenotypes.

##### 3.1.2.1 Child 1 (IV7)

The following are the findings of the physical examination of the 13-year-old son of the proband at the time of the study: Head circumference, 48 cm; chest circumference, 60 cm; height, 127 cm; and weight, 21 kg. The fontanelle had remained open until age 4. Before the age of 2, the toddler had a weak, high-pitched cry resembling a cat’s meow (the cry became normal after that age). His diet consisted of liquid foods because he could not chew solid foods. He had mild nutritional and developmental delays, his physical appearance was characterized by a small head, round face, wide-set eyes, small chin, a high-arched palate, low-set ears, and a normal nose. He had a single transverse palmar crease on both hands, bilateral pes cavus, low muscle tone in all limbs, unstable gait with a wide base, urinary and fecal incontinence, severe intellectual disability, and could not speak or communicate. Brain MRI results suggested corpus callosum dysgenesis, whereas a cardiac ultrasound was normal.

##### 3.1.2.2 Child 2 (IV5)

The physical examination findings of the 35-month-old niece of the proband at the time of the study included: Head circumference, 42 cm; height, 82 cm; and weight, 15 kg. Her vital signs were stable, and the anterior fontanelle was closed. Craniofacial deformities included dolichocephaly, low-set ears, a low and flat nasal bridge, a short and broad nose, forward-tilted nostrils, and a short neck. There were no joint deformities in the limbs, and the physical examinations of the heart, lungs, and abdomen were all normal. The neurological examination revealed appropriate consciousness but slow responses, lack of eye contact, a normal ability to track objects and be amused, absence of strabismus, inability to babble, and incontinence. She could lift her head and sit independently, but could not crawl, had low muscle tone in all limbs, could stand with support but could not walk, and had experienced two seizure-like limb twitches at age 2. Bilateral knee reflexes were symmetrically elicited, and bilateral Babinski signs were negative. The examination yielded normal sensory system tests, and crude hearing and visual responses. Brain MRI results suggested corpus callosum dysgenesis, whereas the cardiac ultrasound was normal.

The parents of IV5 had no phenotypic abnormalities, and they denied consanguinity. The maternal grandparents also were phenotypically normal. There was no history of viral infections during the mother’s pregnancy, nor exposure to toxic substances or radiation.

### 3.2 Genetic testing results

**FIGURE 2:**
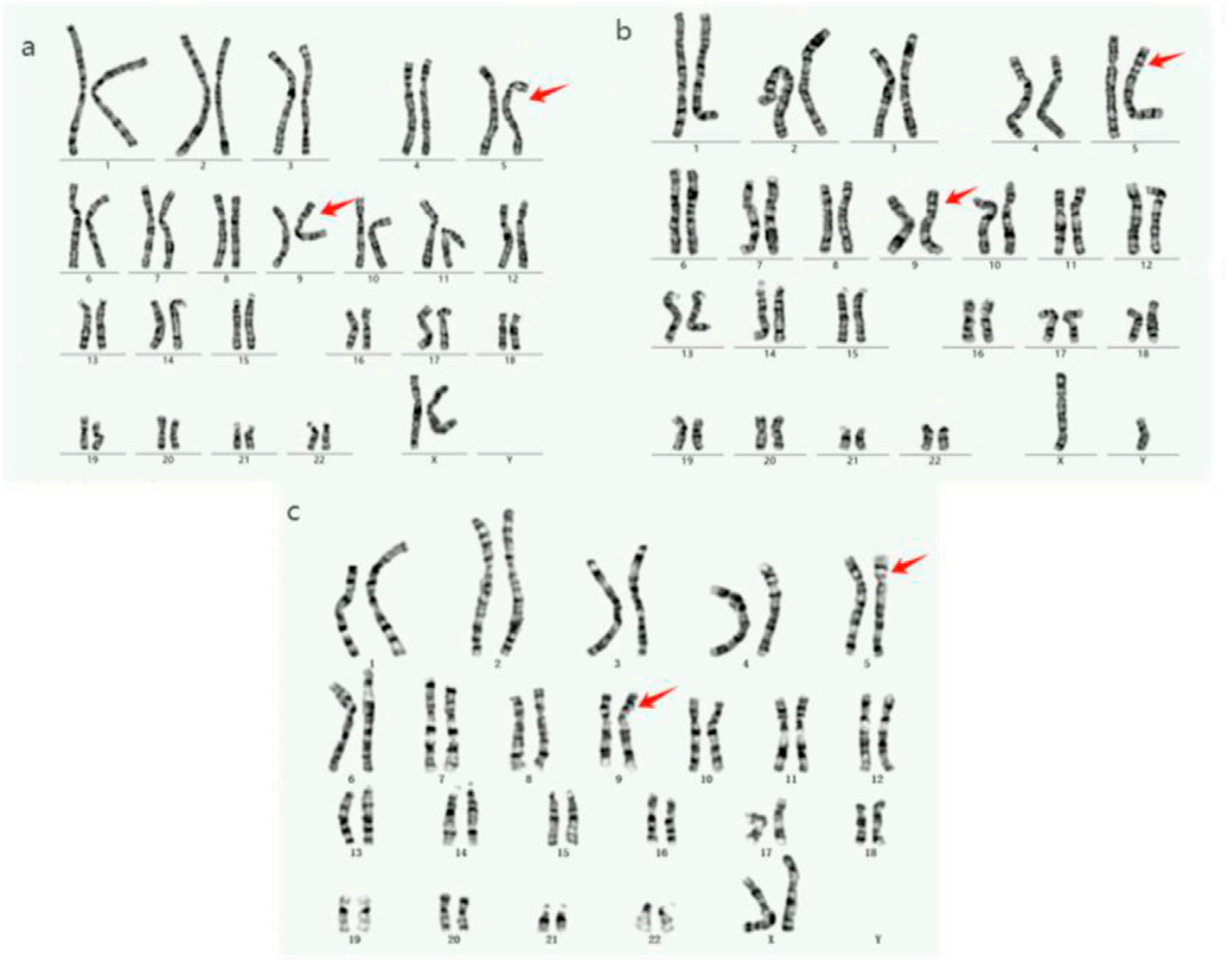
Chromosome karyotype diagram of the proband and family members. **(a)**: The karyotypes of proband III7, father II3, brother III5, and cousin III14 are all 46,XY,der (5)t (5; 9) (p14; p22). **(b)**: The IV7 chromosome karyotype of the proband’s son is 46, XY, der (5)t (5; 9) (p14; p22). **(c)**: The proband’s niece (IV5) has a 46,XX,der (9)t (5; 9) (p15; p24) karyotype.

**FIGURE 3:**
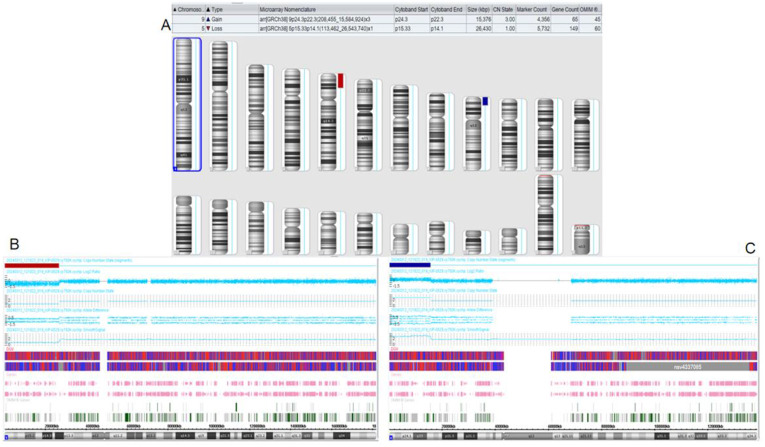
Chromosomal microarray analysis (CMA) results of the son of the proband. **(A)** CMA global picture (deletions are marked in red, duplications are marked in blue) **(B)**: The p15.33-p14.1 fragment in chromosome 5 has a 26.43 Mb single copy deletion. **(C)**: The p24.3-p22.3 segment of chromosome 9 has a three-copy duplication of 15.376 Mb.

**FIGURE 4:**
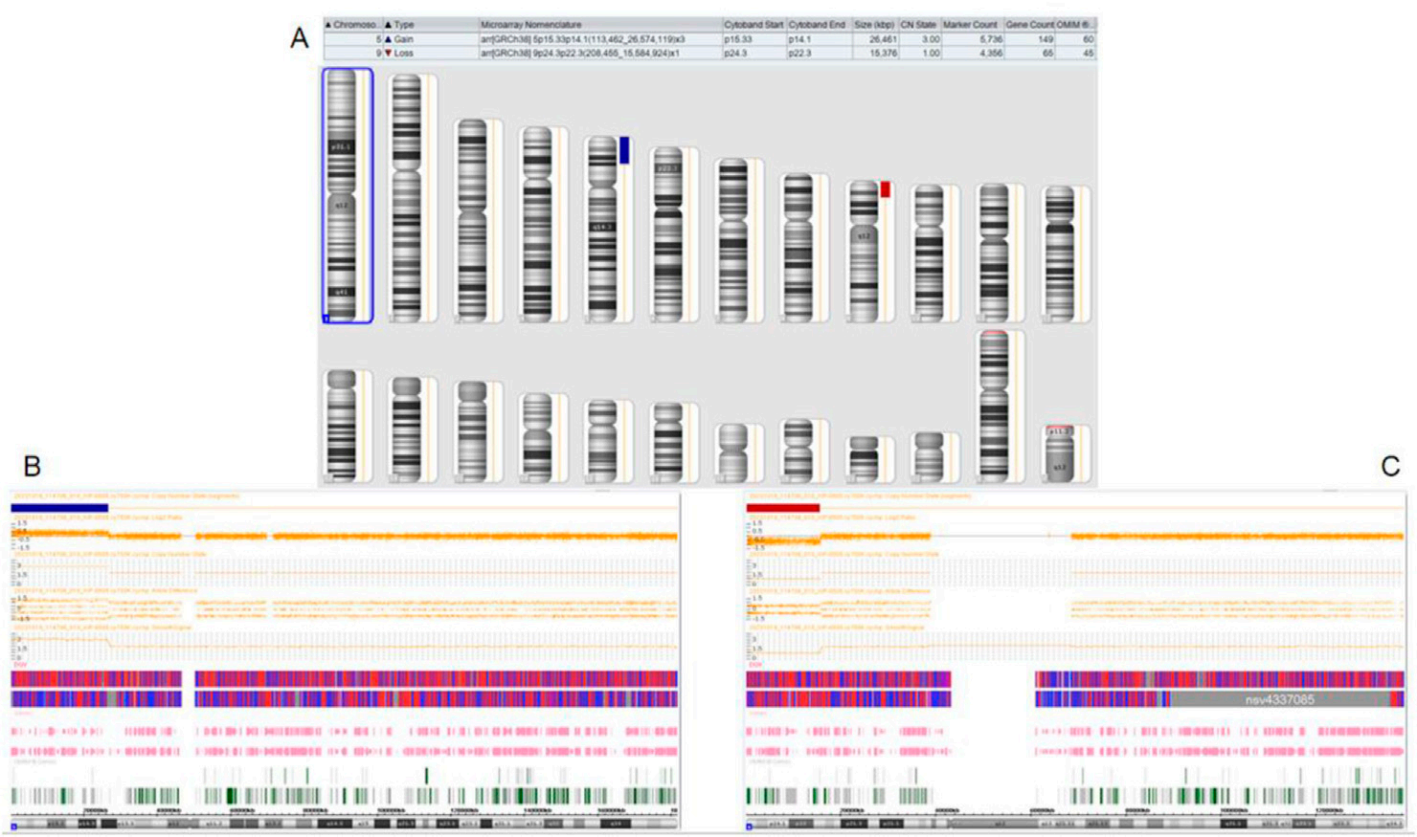
Chromosomal microarray analysis results of the proband’s niece. **(A)**: CMA global picture, (deletions are marked in red and duplications in blue) **(B)**: The p15.33-p14.1 segment of chromosome 5 has a three-copy duplication of 26.461 Mb. **(C)**: The p24.3-p22.3 fragment of chromosome 9 has a single copy deletion of 15.376 Mb.

The proband’s family was originally from Yanshi (Hubei) and consisted of 42 members, 15 participated in the study and six of their offspring developed symptoms. Figure 1 shows the family map of the t (5; 9) (p15; p24) balanced translocation. Figures 2–4 and Table 1 show the chromosomal karyotypes and CNVs of the participating family members. Table 2 shows a summary of functional genes contained in abnormal chromosome segments.

**TABLE 1:** Summary of chromosomes and CMA results of the 13 family members participating in the study.

Family relationship	Sex	Age	Clinical characteristics	Checked items	Results
Mother (Ⅱ4)	female	60	Good health condition	karyotype	46,XX
Father (Ⅱ3)	male	62	Phenotypically normal	karyotype	46,XY,t (5; 9) (p15.1; p24)
Proband (Ⅲ7)	female	35	4 adverse pregnancy outcome	karyotype	46,XY,t (5; 9) (p15.1; p24)
Husband (Ⅲ8)	male	38	Good health condition	karyotype	46,XY
				CytoScan 750K	arr (X,1∼22)×2
Son (Ⅳ7)	male	11	Multiple deformities	karyotype	46,XY,der (5)t (5; 9) (p14; p22)
				CytoScan 750K	arr [GRCh38] 5p15.33p14.1 (113,462_26,543,740)x1,9p24.3p22.3 (208,455_15,584,924)x3
amniotic fluid (Ⅳ9)		16+	No significant ultrasound abnormalities	karyotype	46,XN,add (9)p (22)
				CytoScan 750K	arr [GRCh37]5p15.33p14.1 (130931_26207543)x3 9p24.3p22.3 (239426_15528385)x1
elder brother (Ⅲ5)	male		Phenotypicall normal	karyotype	46,XY,t (5; 9) (p15.1; p24)
Brother’s daughter (Ⅳ5)	female	2	Multiple deformities	karyotype	46,XX,der (9)t (5; 9) (p15.1; p24)
				CytoScan 750K	arr [GRCh38] 5p15.33p14.1 (113,462_26,574,119)x3,9p24.3p22.3 (208,455_15,584,924)x1
Cousin (Ⅲ14)	female	36	1 adverse pregnancy outcome	karyotype	46,XX,t (5; 9) (p15.1; p24)
Cousin’s husband (Ⅲ15)	male	37	Good health condition	karyotype	46,XY
cousin’s amniotic fluid (Ⅳ12)		20+	No significant ultrasound abnormalities	CytoScan 750K	5p15.33p14.1 × 3,9p24.3p22.3 × 1
cousin’s son (Ⅳ11)	male	11	Good health condition	karyotype	46,XY,22pstk+
Cousin (Ⅲ9)	female	28	Good health condition	karyotype	46,XX

**TABLE 2:** Functional genes contained in abnormal chromosome segments carried by the affected children.

Chromesome	Type	Microarray nomenclature	Cytoband start	Cytoband end	Size (kbp)	CN State	Marker count	Gene count	OMIM
5	Loss	arr [GRCh38] 5p15.33p14.1 (113,462_26,543,740)x1	p15.33	p14.1	26,430	1	5,732	149	SDHA (600857), PDCD6 (601057), AHRR (606517), EXOC3 (608186), SLC9A3 (182307), CEP72 (616475), TPPP (608773), BRD9 (618465), TRIP13 (604507), NKD2 (607852), SLC12A7 (604879), SLC6A19 (608893), SLC6A18 (610300), TERT (187270), CLPTM1L (612585), SLC6A3 (126455), LPCAT1 (610472), MRPL36 (611842), NDUFS6 (603848), IRX4 (606199), LSINCT5 (615764), IRX2 (606198), IRX2-DT (610522), IRX1 (606197), ADAMTS16 (607510), ICE1 (617958), MED10 (612382), UBE2QL1 (615832), LINC01018 (616385), NSUN2 (610916), SRD5A1 (184753), TENT4A (605198), ADCY2 (103071), FASTKD3 (617530), MTRR (602568), SEMA5A (609297), TAS2R1 (604796), ATPSCKMT (618568), CCT5 (610150), CMBL (613379), MARCHF6 (613297), ROPN1L (611756), DAP (600954), CTNND2 (604275), LINC01194 (617097), DNAH5 (603335), TRIO (601893), OTULIN (615712), ANKH (605145), FBXL7 (605656), MARCHF11 (613338), ZNF622 (608694), RETREG1 (613114), MYO10 (601481), BASP1 (605940), CDH18 (603019), CDH12 (600562), PMCHL1 (176793), PRDM9 (609760), CDH10 (604555)
9	Gain	arr [GRCh38] 9p24.3p22.3 (208,455_15,584,924)x3	p24.3	p22.3	15,376	3	4,356	65	DOCK8 (611432), KANK1 (607704), DMRT1 (602424), DMRT3 (614754), DMRT2 (604935), SMARCA2 (600014), VLDLR (192977), KCNV2 (607604), PUM3 (609960), RFX3 (601337), GLIS3 (610192), SLC1A1 (133550), PLPP6 (611666), CDC37L1 (610346), AK3 (609290), RCL1 (611405), MIR101-2 (612512), JAK2 (147796), INSL6 (606414), INSL4 (600910), RLN2 (179740), RLN1 (179730), PLGRKT (618444), CD274 (605402), PDCD1LG2 (605723), RIC1 (610354), ERMP1 (611156), MLANA (605513), IL33 (608678), TPD52L3 (617567), UHRF2 (615211), GLDC (238300), KDM4C (605469), DMAC1 (617261), PTPRD (601598), TYRP1 (115501), LURAP1L (616130), MPDZ (603785), NFIB (600728), ZDHHC21 (614605), CER1 (603777), FREM1 (608944), TTC39B (613574), SNAPC3 (602348), PSIP1 (603620)
5	Gain	arr [GRCh38] 5p15.33p14.1 (113,462_26,574,119)x3	p15.33	p14.1	26,461	3	5,736	149	SDHA (600857), PDCD6 (601057), AHRR (606517), EXOC3 (608186), SLC9A3 (182307), CEP72 (616475), TPPP (608773), BRD9 (618465), TRIP13 (604507), NKD2 (607852), SLC12A7 (604879), SLC6A19 (608893), SLC6A18 (610300), TERT (187270), CLPTM1L (612585), SLC6A3 (126455), LPCAT1 (610472), MRPL36 (611842), NDUFS6 (603848), IRX4 (606199), LSINCT5 (615764), IRX2 (606198), IRX2-DT (610522), IRX1 (606197), ADAMTS16 (607510), ICE1 (617958), MED10 (612382), UBE2QL1 (615832), LINC01018 (616385), NSUN2 (610916), SRD5A1 (184753), TENT4A (605198), ADCY2 (103071), FASTKD3 (617530), MTRR (602568), SEMA5A (609297), TAS2R1 (604796), ATPSCKMT (618568), CCT5 (610150), CMBL (613379), MARCHF6 (613297), ROPN1L (611756), DAP (600954), CTNND2 (604275), LINC01194 (617097), DNAH5 (603335), TRIO (601893), OTULIN (615712), ANKH (605145), FBXL7 (605656), MARCHF11 (613338), ZNF622 (608694), RETREG1 (613114), MYO10 (601481), BASP1 (605940), CDH18 (603019), CDH12 (600562), PMCHL1 (176793), PRDM9 (609760), CDH10 (604555)
9	Loss	arr [GRCh38] 9p24.3p22.3 (208,455_15,584,924)x1	p24.3	p22.3	15,376	1	4,356	65	DOCK8 (611432), KANK1 (607704), DMRT1 (602424), DMRT3 (614754), DMRT2 (604935), SMARCA2 (600014), VLDLR (192977), KCNV2 (607604), PUM3 (609960), RFX3 (601337), GLIS3 (610192), SLC1A1 (133550), PLPP6 (611666), CDC37L1 (610346), AK3 (609290), RCL1 (611405), MIR101-2 (612512), JAK2 (147796), INSL6 (606414), INSL4 (600910), RLN2 (179740), RLN1 (179730), PLGRKT (618444), CD274 (605402), PDCD1LG2 (605723), RIC1 (610354), ERMP1 (611156), MLANA (605513), IL33 (608678), TPD52L3 (617567), UHRF2 (615211), GLDC (238300), KDM4C (605469), DMAC1 (617261), PTPRD (601598), TYRP1 (115501), LURAP1L (616130), MPDZ (603785), NFIB (600728), ZDHHC21 (614605), CER1 (603777), FREM1 (608944), TTC39B (613574), SNAPC3 (602348), PSIP1 (603620)

### 3.3 Enrichment analysis results

**FIGURE 5:**
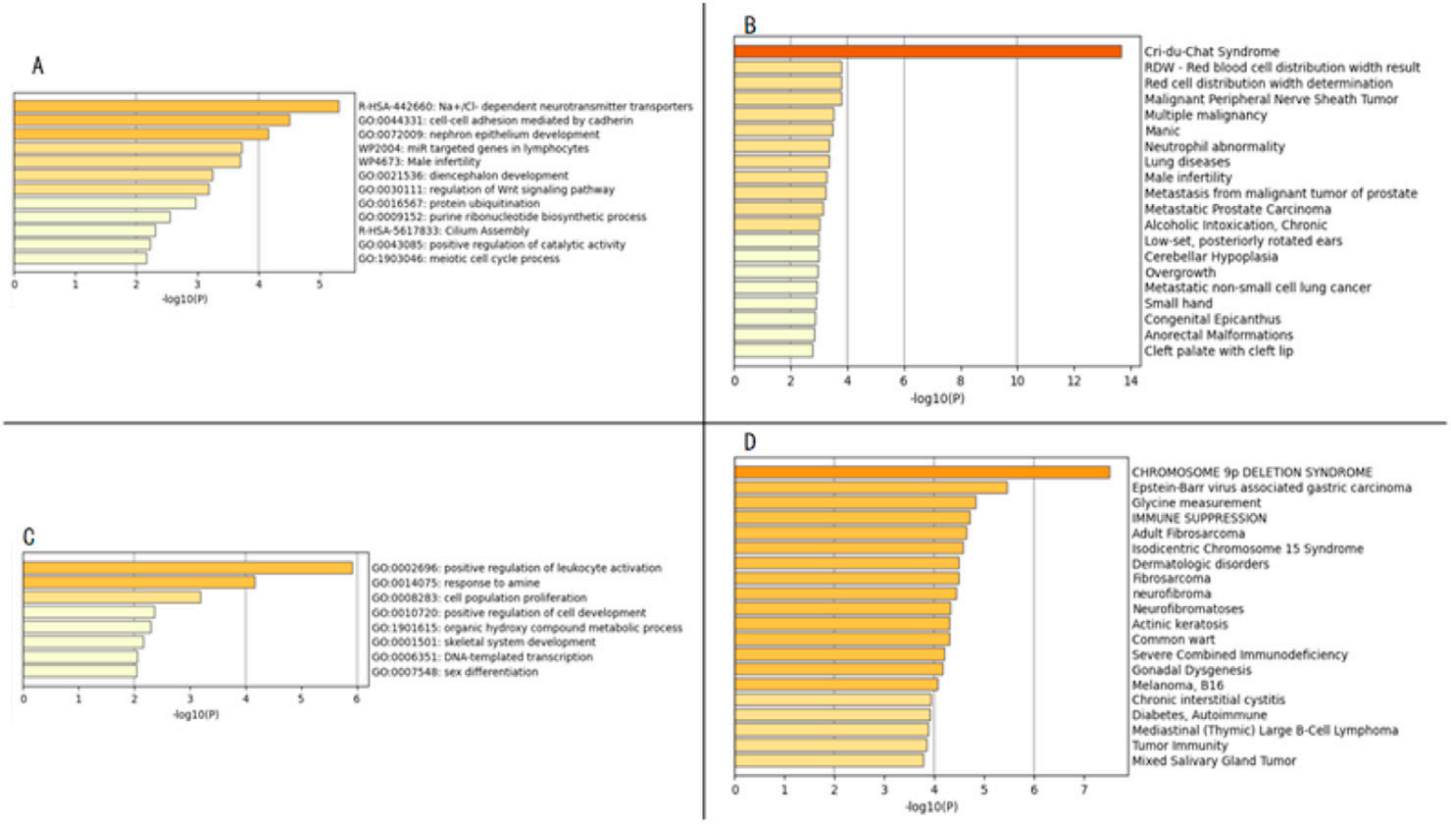
Panels **(A,C)** display the enrichment analysis results of gene functions from the Gene Ontology database, Reactome database, and Wikipathway database; Panels **(B,D)** show the enrichment analysis results of related diseases from the DisGeNET database.

Using the Metascape database for GO enrichment analysis of the region containing 60 OMIM genes from 5p15.33p14.1 revealed the potential molecular mechanisms of the disease. The results showed that OMIM genes in the 5p15.33p14.1 region are mainly enriched in Na+/Cl-dependent neurotransmitter transporters, cell-cell adhesion mediated by cadherin, nephron epithelium development, and other signaling pathways (Figure 5A). Disease enrichment analysis showed that genes in this region are mainly associated with Cri-du-chat syndrome (Figure 5B) . Cri-du-chat syndrome is closely related to developmental abnormalities, neurological defects, and craniofacial malformations. Enrichment analysis supports the involvement of molecular mechanisms related to Wnt signaling, neurotransmitter transport, ubiquitination pathways, particularly through disease-gene associations from DisGeNET and GO functional enrichment. These results provide clues for revealing the molecular network of the disease and guide future research.

Using the Metascape database, GO enrichment analysis of 45 OMIM genes located in the 9p24.3-p22.3 region was performed. The results showed that OMIM genes in the 9p24.3-p22.3 region are mainly enriched in signaling pathways such as positive regulation of leukocyte activation, response to amine, cell population proliferation, positive regulation of cell development, etc. (Figure 5C). Disease enrichment analysis revealed that genes in this region are mainly associated with Chromosome 9p deletion syndrome (Figure 5D). This study, through multidimensional bioinformatics analysis, not only clarified the core biological functions of genes in the 9p24.3-p22.3 region, but also revealed their potential association mechanisms with major diseases, providing important theoretical basis and directional guidance for subsequent gene function validation, molecular mechanism research, and clinical translation.

## 4 Discussion

Balanced translocation carriers have the opportunity to produce phenotypically normal offspring, but they are at a higher risk of recurrent miscarriages and offspring with chromosomal abnormalities. This study reports a rare familial balanced translocation pedigree, particularly noting that the offspring can suffer from Cri-du-chat syndrome, which suggests a potential new genetic model for this syndrome. It provides novel insights for genetic counseling and prenatal diagnosis in patients with adverse pregnancy outcomes before attempting another pregnancy.

In this study, the proband, the proband’s father, the proband’s brother, and cousin are all carriers of t (5; 9) (p14; p22) balanced translocation genes, and their families have adverse pregnancy outcomes. Specifically, the proband’s surviving child (IV7) was diagnosed with 5p deletion/9p duplication syndrome. The copy number loss in the 5p15.33p14.1 region was deemed a pathogenic CNV, involving 60 OMIM genes. This segmental loss may lead to Cri-du-chat syndrome (CdCS). Mainardi et al. found that large deletions and unbalanced translocations in CdCS are closely associated with severe neurologic impairment and congenital malformations (Mainardi et al., 2001). Other studies have also confirmed that large deletions in the short arm of chromosome five can result in severe intellectual disabilities, significant speech delay, behavioral problems, and hypotonia (Espirito et al., 2016). In this case, the patient exhibited typical intellectual disabilities and hypotonia, consistent with literature reports.

Copy number variations in the 9p24.3-p22.3 region were identified in the patient (Ⅳ7), currently classified as clinically unclear CNVs, including 45 OMIM pathogenic genes such as *NFIB*. It is worth noting that previous studies have suggested that such complex rearrangements can result in overlapping phenotypes (Lincoln-de-Carvalho et al., 2011). The duplication in the 9p region involves the *NFIB* gene, the abnormality of which has been confirmed to be associated with acquired megalencephaly with intellectual disability, mainly characterized by intellectual disability, hypotonia, and seizures (Schanze et al., 2018; Barrus et al., 2020; Rao and Goel, 2020). Clinical correlation analysis indicates that the duplication of the *NFIB* gene in this case may synergistically exacerbate neurodevelopmental abnormalities such as intellectual disability and hypotonia.

Approximately 80% of copy number deletion variations in the 5p region are caused by *de novo* mutations, with only 10%–15% of cases inherited from parents carrying balanced translocations of this segment (Mainardi et al., 2006). For instance, Nur Kırman et al. (Kırman et al., 2025) reported a case of a maternally derived balanced translocation between chromosomes four and 5 (t (4; 5) (q27; pter)), leading to a 5p15.33-p14.3 deletion and a 4q26-q35.2 duplication in the offspring, clinically manifesting as a composite phenotype of 5p deletion syndrome (cat-like cry, distinctive facial features, hypotonia, etc.) and 4q duplication syndrome (congenital heart disease, limb abnormalities, etc.). Another study reported paternal gonadal mosaicism resulting in three siblings all carrying a 5p29-Mb deletion and a 19q4.7-Mb duplication (Alkaya et al., 2020); furthermore, Li et al. (Zhang et al., 2020) documented a maternally derived t (4; 5) (q33; p15) leading to a 5p15.2-p15.33 deletion and a 4q32.3-q35.2 duplication in three consecutive pregnancies. The 5p deletion in this pediatric case was inherited from the mother who carried a balanced translocation of this segment. Notably, no reports of t (5; 9) (p14; p22) translocation have been retrieved to date, indicating that this translocation is extremely rare. Based on the clinical manifestations of this case, the 5p deletion remains the core pathogenic factor, but the 9p duplication may contribute to phenotypic modification through abnormal expression of dosage-sensitive genes. This case provides new evidence for the clinical-genetic correlation study of 5p-/9p + complex chromosomal disorders, emphasizing the necessity of multi-locus collaborative analysis in the diagnosis of complex cases. It also suggests that when facing patients with Cri-du-chat syndrome, the possibility of parental balanced translocation should be considered to better guide reproductive counseling.

The niece of the proband (Ⅳ5) and the son of the proband (Ⅳ7) have the opposite pattern of missing regions, indicating a 5p duplication/9p deletion syndrome patient. The pathogenic copy number variation (CNV) involving the 5p15.33p14.1 region, encompassing 60 OMIM genes, was identified in this case, which is consistent with the phenotype of neurological developmental disorders and hypotonia reported in the literature (Riordan et al., 2002; Sheen et al., 2003; Baialardo et al., 2003). The deletion in the 9p24.3-p22.3 region of chromosome 9 has also been identified as a pathogenic CNV, closely associated with 9p deletion syndrome (OMIM: 158170) (Banerjee et al., 2020). In addition to the typical triad of Chromosome 9p deletion syndrome (intellectual disability, distinctive facial features, and hypotonia), this case further revealed agenesis of the corpus callosum as a diagnostic radiological sign. A study by Spazzapan et al. (2016) showed that over 50% of patients with this syndrome exhibit corpus callosum malformations, highlighting this feature as an important expansion of the core phenotype spectrum. Although Chromosome 9p deletion syndrome is typically a *de novo* mutation in around 99% of cases, this case represents the rare 1% that is familial in origin (Sivasankaran et al., 2016), underscoring the key value of multi-generational tracking in elucidating the genetic patterns of chromosomal rearrangements.

This study reveals that carriers of balanced translocations have a significant chromosome bias in reproductive risks, with an increased 3:1 segregation ratio when involving chromosomes with centromeres or breakpoints near centromeres (such as the familial t (5; 9) (p14; p22)) (Yu et al., 2023; Tonyan et al., 2024). Female carriers are more likely to produce unbalanced gametes. This abnormal segregation pattern results in offspring with dual inherited risks: either presenting as 5p deletion/9p duplication (IV7) or forming a complementary 5p duplication/9p deletion (IV5) configuration.

This study presents a rare familial t (5; 9) (p14; p22) balanced translocation inheritance pattern, which is globally rare in the reports of complex chromosomal rearrangement abnormalities. This case provides multiple implications for clinical practice. When encountering couples with a history of recurrent adverse pregnancy outcomes, it is recommended to actively undergo genetic testing to timely identify carriers of balanced translocations, and to actively provide genetic counseling and prenatal diagnosis, which are of great significance. Through systematic genetic analysis and prenatal diagnosis, the incidence of offspring chromosomal abnormalities can be effectively reduced, thereby improving reproductive outcomes and enhancing population quality.

## Data Availability

The datasets presented in this study can be found in online repositories. The names of the repository/repositories and accession number(s) can be found in the article/supplementary material.

## Abbreviations

CMA, hromosomal microarray analysis; CNVs, copy number variants.
